# Effectiveness of fractional erbium–YAG laser, microneedling, platelet-rich plasma in localized stable vitiligo patients: randomized clinical trial

**DOI:** 10.1007/s00403-024-03035-8

**Published:** 2024-06-15

**Authors:** Soheir Abdel-Hamid, Hassan M. Ibrahim, Aya M. Hameed, Eisa M. Hegazy

**Affiliations:** 1 Department of Dermatology, Venereology, and Andrology, Faculty of Medicine, South Valley University, Qena, Egypt; 2 Dermatology, Venereology, and Leprosy Hospital, Qena, Egypt

**Keywords:** Er–YAG laser, Microneedling, Platelet-rich plasma, VASI score, Vitiligo

## Abstract

Vitiligo is considered an autoimmune disease, and its treatment is challenging. We assessed and compared the effect of fractional erbium:yttrium–aluminum–garnet (Er:YAG) laser-assisted delivery of platelet-rich plasma versus microneedling (Mn) with platelet-rich plasma (PRP) in enhancing skin repigmentation in localized stable vitiligo patients. In total, 40 patients with localized stable vitiligo were selected in a random manner into two similar groups (20 each). Group (A) was subjected to fractional Er:YAG laser combined with platelet-rich plasma and Group (B) was subjected to microneedling combined with platelet-rich plasma. The procedure was repeated every 2 weeks for up to 6 months. Each individual was assessed clinically utilizing Vitiligo Area Scoring Index (VASI). Fractional Er:YAG + PRP group achieved better pigmentation100% (excellent 30%, very good 15%, good 30% and satisfactory 25%) which is comparable to Mn + PRP where 80% of cases demonstrate repigmentation (20% very good, 10% good and 50% mild). When comparing the VASI scores for both groups after therapy to the baseline VASI, there was a statistically significant decrease [p = 0.001 for group(A) and 0.003 for group(B)]. Regarding the treatment side effects, there was significantly (p = 0.048) side effects among cases treated with microneedling group(B) (25%) than those fractional Er:Yag laser therapy group(A) (5%). Both forms of therapy demonstrated induction of repigmentation of vitiligo, but fractional Er:YAG laser efficacy is greater when combined with platelet-rich plasma.

Clinical trials.gov identifier: NCT05511493.

**Supplementary Information:**

The online version contains supplementary material available at 10.1007/s00403-024-03035-8.

## Introduction

Vitiligo is a skin pigmentation disorder caused by the selective degradation of melanocytes [1]. Affects 0.5–2.0% of the global population [2]. Vitiligo is a complex disease whose pathogenesis caused by a combination of hereditary factors, metabolic variables associated with oxidative stress, melanocyte adherence to the epithelium and innate and adaptive immunity that ultimately leads to melanocytes destruction [3]. Though several therapy approaches have been identified for repigmentation, the complex and polygenic character of vitiligo pathogenesis affects unpredictable outcome responses and disappointing results. Therefore, rather than using monotherapy, clinicians are treating patients with combination medicines to obtain greater repigmentation [4]. Platelet-rich plasma is an autologous therapeutic modality that has been developed for many dermatological conditions, including vitiligo [5]. Microneedling, a therapeutic technique that causes physical damage to the skin by puncturing the epidermis with a needle. This triggers the wound healing cascade. It improves topical therapy absorption outside of the thick stratum corneum [6]. Laser-assisted drug delivery (LADD) creates certain controllable microscopic ablation zones (MAZs) surrounded by intervening regions of intact skin [7]. Of them, fractional erbium:yttrium–aluminum–garnet (Er:YAG) has drawn the most interest because of its greater medical benefits and less side effects. This is mostly because it results in less thermal damage and speeds up healing [8].

## Patients and methods

### Study design and participants

The South Valley University Outpatient Clinic’s Dermatology, Venerology, and Andrology Department conducted this prospective randomized clinical trial (RCT) not blinded during the period of May 2023 to November 2023. Individuals with stable localized vitiligo in both sexes: stable patches (should not change in size or pigmentation for a minimum of 3 months). Age between 10 and 60 years were included. Patients with acute or chronic infections, Koebner phenomenon, pregnant or lactating women, patients with a history of altered or abnormal fibroblast function, such as collagen disorders or myelofibrosis, patients on anticoagulants or antiplatelet medications, and patients with blood or platelet abnormalities were excluded. Prior to the trial, all of the patients had not used any systemic or local medications for at least 2 months. Every patient had a comprehensive history taking, as well as a general and dermatological examination. We adjusted sample size to achieve 80% power and a 5% confidence level of significance (type 1 error).

### Randomization and masking

Patients were randomly allocated (1:1) to receive either fractional Er:YAG laser-assisted delivery of PRP or versus Microneedling with PRP. Randomization was performed using closed envelopes. Patients and data interpreters were blinded to the trial drug assignment, but not researchers or medical professionals.

### Sample size calculation

We utilized the G*power 3 algorithm [9] to calculate the sample size. An estimated minimum sample size of 40 participants was required to find a size of 0.3 in the association between the effects of fractional Er:YAG laser-assisted delivery of platelet-rich plasma versus microneedling with PRP in improving skin repigmentation in localized stable vitiligo patients with an error possibility of 0.05 and an 80% power. Each participant was allocated to one of the two collections. Thus, there were two groups in the study: in the first, 20 vitiligo patients received PRP delivery assisted by a fractional Er:YAG laser, while in the second, 20 vitiligo patients received PRP delivery via microneedling.

### Ethical considerations

The Faculty of Medicine Ethics Committees at South Valley University gave their approval for the current study, under approval number SVU, MED, DVA021,22,8,426. The Institutional Review Board-Ethics committee of the South Valley University, Faculty of Medicine gave its clearance for the study, which was conducted in compliance with the Declaration of Helsinki’s guiding principles. The trial was listed on the website for clinical trial registration (https://clinicaltrials.gov/, Identifier: NCT05511493). Before beginning, each participant had to put their signature on a consent form. It was evident that informed consent was obtained and provided information on the goals, methodology, advantages and dangers of the study in addition to the participants’ unrestricted right to join or withdraw at any time.

### History and clinical examination

Comprehensive medical history and disease specifics, including initiation, progression, duration, location of lesions, prior treatment and familial predisposition to vitiligo or other autoimmune disorders. Full clinical examination and adequate dermatologic examination with special consideration to skin Fitzpatrick phototypes, hair, nails, oral mucosa and anatomical sites of vitiligo. Before beginning treatment, there was a minimum 3-month washout period during which none of the patients received any systemic or local medication.

### Treatment protocol

The following treatment protocols were followed for each group: Group A was subjected to fractional Er: YAG laser and treated areas are covered with PRP. Group B was treated with PRP after receiving microneedling using an electronic dermapen instrument (Dr. Pen Derma Pen Ultima A6®).

### PRP preparation

Blood was drawn and placed in tubes with an anticoagulant formula (Na citrate solution). The blood was citrated and centrifuged at 3000 rpm for 7 min. The plasma was then taken up, carrying a buffy covering with platelets and leukocytes. The second round of centrifugation for a duration of 5 min at 4000 rpm was done. The platelet-rich plasma rose to the top while the platelet-poor plasma pooled at the bottom of the pellet [10].

### Steps of the procedure

The affected area was cleaned with a 70% alcohol solution and then betadine surgical solution. A topical anesthetic termed pridocaine cream was used before the procedure to the intended site and left in place for 30 min with an occlusive bandage. Group(A): patients were subjected to a fractional Er:YAG laser (FotonaXs Dynamis, Slovenia) operating in short pulse mode (SP) at a power of 1400 mJ, pixel 1, and a spot size of 7 mm in diameter. Following this, PRP was applied to the treated areas. Group (B): the utilization of an automated microneedling, which is the Dr Pen Derma Pen Ultima A6®. The derma pen may reach varying depths in the skin, up to 0.25 to 0.5 mm, but not below. It proceeded from the perilesional regions in a circular motion toward the depigmented center, traversing the vitiligo region vertically until localized bleeding began [11]. The treated areas were then covered with PRP. For 6 months, this process was carried out once every 2 weeks.

### Dermatological evaluation of the included patients

Before, monthly and after treatments, standardized high-resolution digital photos taken with the same camera settings (Samsung) were recorded. Clinical re-pigmentations according to Physician’s Global Assessment (PGA) [12]. G0 (poor: no repigmentation), G1 (satisfactory: < 25% repigmentation), G2 (good: 25–50% repigmentation), G3 (very good: 50–75% repigmentation), and G4 (excellent: > 75% repigmentation), and are the five categories. Vitiligo Area Scoring Index (VASI): the degree of depigmentation inside each determined patch is multiplied by the area of vitiligo in hand units to determine the VASI for each anatomical location [13]. Evaluation of repigmentation pattern includes an assessment of its marginal, perifollicular, diffuse, combined and mixed characteristics. Patient satisfaction is recorded as Grade 4 (very satisfied), Grade 3 (satisfied), Grade 2 (neutral) and Grade 1 (dissatisfied). Side effects: every patient should follow-up 1 week following each session to detect any side effects as soon as possible.

## Statistical analysis

The data underwent a process of verification, coding, and analysis using IBM-SPSS 24.0 (IBM-SPSS Inc., Chicago, IL, USA). Descriptive statistics refer to the numerical measures that summarize and describe the main characteristics of a dataset. The statistical measures of means, standard deviations, medians, ranges, frequency, and percentages were computed. Statistical significance testing: the statistical tests employed to assess the disparity in frequency distributions among several groups were the Chi-square test, Fisher’s exact test, and the Monte Carlo exact test, as deemed suitable. A p value is deemed significant if it is equal to or less than 0.05. The studied SNP followed the Hardy Weinberg (HW) equation [14, 15].

## Results

### Clinical characteristic of studied groups

Both groups were matched for age (p = 0.978) i.e., mean age of group(A) was 25.9 ± 2.5 years and group(B) was 26.0 ± 2.6 years. Regarding sex, both groups were matched (p = 0.736) i.e., male/female ratio was 6/14 for group(A) and 7/13 for group(B). Both groups were matched for family history of disease (p = 1.000), and 15% (n = 3) of both groups had positive family history. Also, there was insignificant difference (p = 0.063) in the duration of disease between groups. Moreover, both groups were matched (p = 1.000) in the distribution of skin phenotype according to Fitzpatrick scale among groups. In other words, skin phenotype distribution in both groups was as follows: skin type III, IV and V was 3 (15%), 15 (75%) and 2 (10%) (Table 1).

**Table 1:** Clinical characteristics of the studied groups

	Group A (n = 20)	Group B (n = 20)	*p*-Value
Age (Mean ± SD)	25.90 ± 2.5	26.00 ± 2.6	= 0.978*
Sex			= 0.736**
Male	6 (30%)	7 (35%)	
Female	14 (70%)	13 (65%)	
Family history			= 1.000*
No	17 (85%)	17 (85%)	
Yes	3 (15%)	3 (15%)	
Disease duration/years			= 0.063**
Mean ± SD	3.70 ± 2.1	5.15 ± 2.8	
Median (Range)	3 (1—11)	5 (2—12)	
Fitzpatrick scale skin phenotype			= 1.000*
III	3 (15%)	3 (15%)	
IV	15 (75%)	15 (75%)	
V	2 (10%)	2 (10%)	

*T-test was used to compare the mean difference between groups

**Chi-square test was used to compare the Frequency between groups

### Sites of lesions, repigmentation patterns

Regarding the site of lesions, group(A) had insignificantly (p = 0.525 and 0.548) lower percentage of lower limb, neck and trunk affection (40% and 5%) compared with group-B (50% and 10%). On the other hand, group(A) had insignificantly (p = 0.197 and 0.376) higher percentage of upper limb and face affection (50% and 20) compared with group-B (30% and 10%). Moreover, re-pigmentation was significantly (p = 0.038) not present in about one-fifth (n = 4) of group(B) vs no case in group(A). Also, distribution of re-pigmentation pattern was comparable for the two groups i.e., diffuse (p = 0.744), combined and marginal (p = 0.465) and peri-follicular (p = 0.548) (Table 2).

**Table 2:** Sites of lesions, repigmentation patterns

	Group A (n = 20)	Group B (n = 20)	*p*-Value
Sites of lesion			
Upper limbs	6 (30%)	4 (20%)	= 0.197*
Lower limbs	8 (40%)	10 (50%)	= 0.525*
Face	4 (20%)	2 (10%)	= 0.376**
Neck	1 (5%)	2 (10%)	= 0.548***
Trunk	1 (5%)	2 (10%)	= 0.548***
Re-pigmentation Pattern			
No	0 (0%)	4 (20%)	= 0.038***
Diffuse	8 (40%)	7 (35%)	= 0.744*
Combined	6 (30%)	4 (20%)	= 0.465*
Marginal	6 (30%)	4 (20%)	= 0.465*
Peri-follicular	1 (5%)	2 (10%)	= 0.548*

*Chi-square test was used to compare the frequency between groups

**Mann–Whitney U-test was used to compare the median between groups

***Fisher’s exact test was used to compare the frequency between groups

### Repigmentation response and patients’ satisfaction

**Fig. 1:**
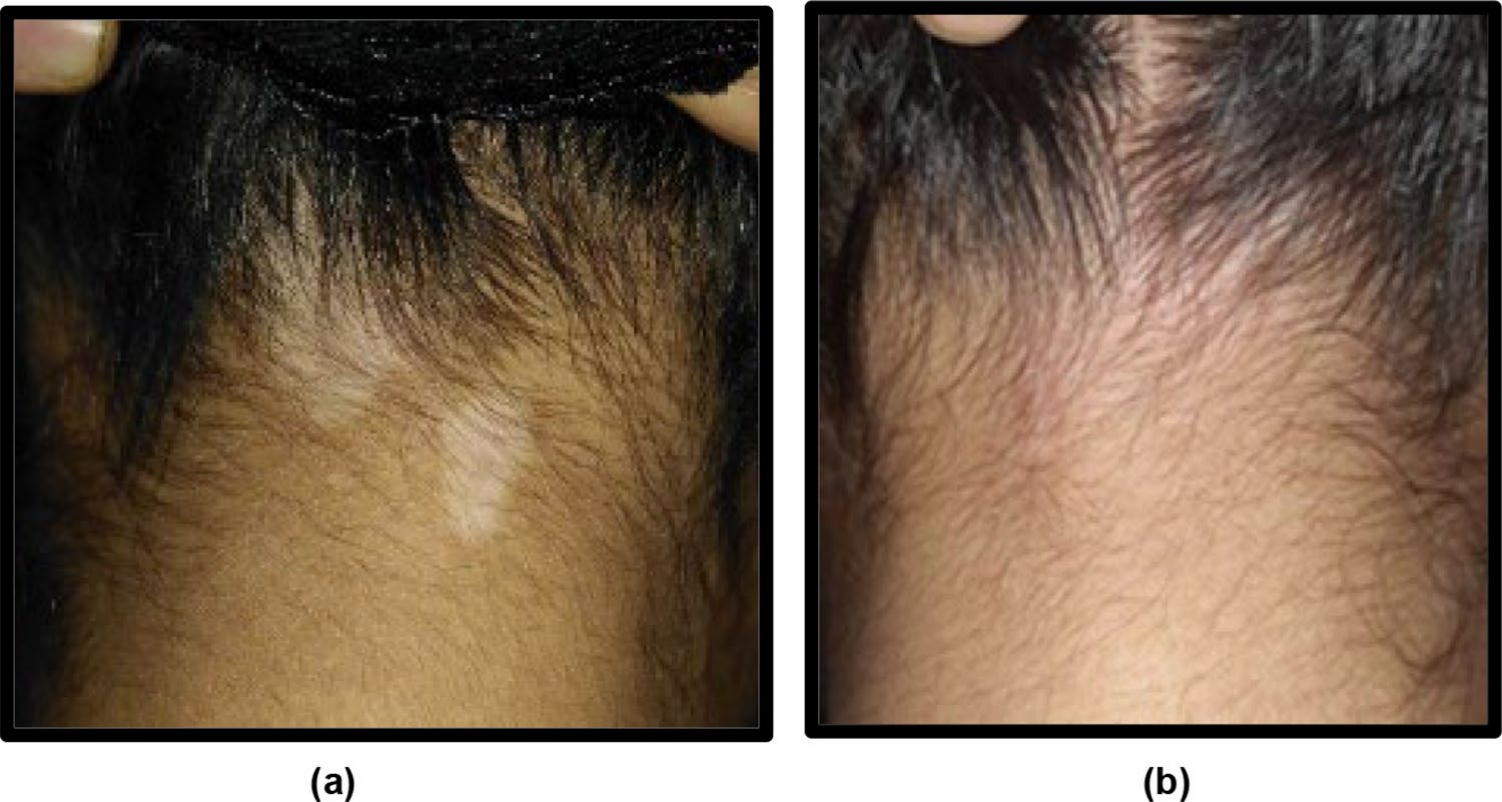
Neck of 19-year-old female patient from group A: **a** before treatment, **b** after 6 months of treatment fractional Er:YAG laser + PRP of showing G4 repigmentation

**Fig. 2:**
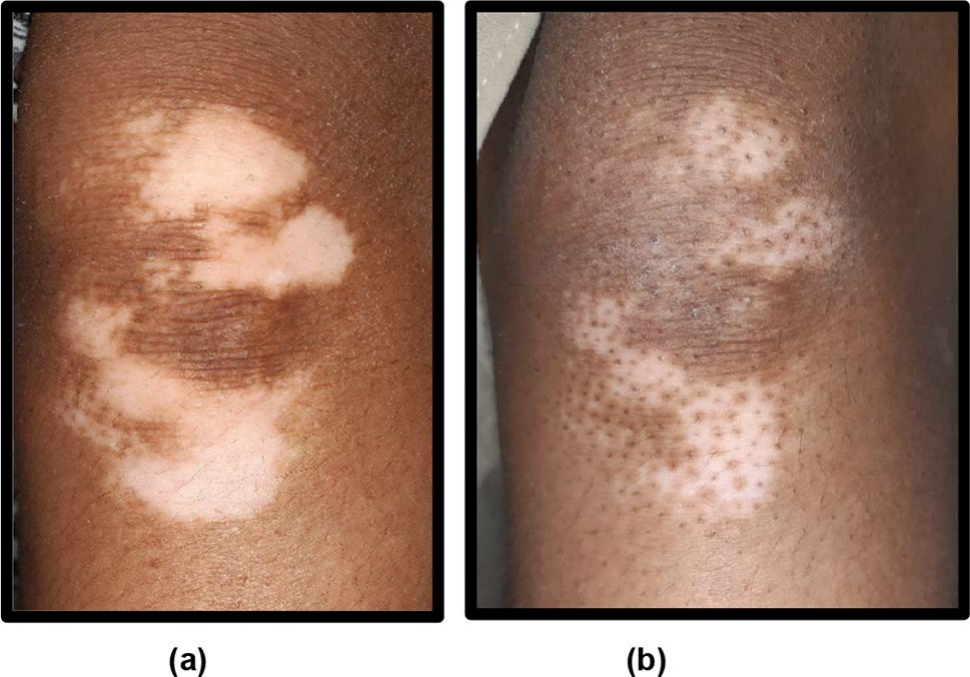
Right knee of 23-year-old male patient from group B: **a** before treatment, **b** after 6-month treatment with microneedling + PRP showing G3 repigmentation

There was significant difference (p = 0.002) between both treatment modalities for the degree of re-pigmentation. In other words, excellent/very good/good improvement was observed in 75% [30% (n = 6), 15% (n = 3) and 30% (n = 6)] in group(A) (Fig. 1) compared with 30% [0% (n = 0), 20% (n = 4) and 10% (n = 2)] in group(B) (Fig. 2). In contrast, satisfactory/poor improvement was observed in 25% [25% (n = 5) and 0% (n = 0)] in group(A) compared with 70% [50% (n = 10), and 20% (n = 4)] in group(B). Furthermore, there was significant difference (p = 0.005) between both treatment modalities for the degree of patients’ satisfaction i.e., highly satisfied/satisfied was observed in 45% [30% (n = 6) and 15% (n = 3)] in group(A) than 15% [0% (n = 0) and 15% (n = 3)] in group(B). In contrast, neutral/dissatisfied was observed in 55% [30% (n = 6) and 25% (n = 5)] in group(A) than 85% [10% (n = 2), and 75% (n = 15] in group(B) (Table 3).

**Table 3:** Repigmentation response and patients’ satisfaction

	Group A (n = 20)	Group B (n = 20)	*p*-Value
Degree of re-pigmentation			= 0.002*
Excellent	6 (30%)	0 (0%)	
Very good	3 (15%)	4 (20%)	
Good	6 (30%)	2 (10%)	
Satisfactory	5 (25%)	10 (50%)	
Poor	0 (0%)	4 (20%)	
Patients’ satisfaction			= 0.005*
Highly satisfied	6 (30%)	0 (0%)	
Satisfied	3 (15%)	3 (15%)	
Neutral	6 (30%)	2 (10%)	
Dissatisfied	5 (25%)	15 (75%)	

*Monte Carlo exact test was used to compare the frequency between groups

### Effect of treatment on VASI score, side effects between groups

Both groups showed no significant difference regarding mean VASI score at baseline (0.35 ± 0.1 vs 0.27 ± 0.1, p = 0.571) and after treatment (0.19 ± 0.1 vs 0.23 ± 0.1, p = 0.725). Moreover, when comparing the VASI scores for both groups after therapy to the baseline VASI, there was a statistically significant decrease (p = 0.001 for group(A) and 0.003 for group(B). Likewise, there was significant difference for the interaction between time and mode type (p = 0.017), i.e., group(A) had better result for the VASI score (reduction of 16%) compared with group(B) (reduction of 4%) Regarding the treatment side effects, there was significantly (p = 0.048) higher rates of side effects among cases treated with microneedling group(B) (25%) than those treated with Er:Yag laser group(A) (5%) (Table 4).

**Table 4:** Effect of treatment on VASI score, side effects between groups

	Group A (n = 20)	Group B (n = 20)	*p*-Value
VASI score			
Baseline	0.35 ± 0.09	0.27 ± 0.06	= 0.571*
After treatment	0.19 ± 0.08	0.23 ± 0.07	= 0.725*
P-value**	= 0.001	= 0.003	= 0.017***
Side effects			
No	19 (95%)	15 (75%)	= 0.048**
Yes	1 (5%)	5 (25%)	

*Student t-test was used to compare mean between groups

**Paired sample t-test was used to compare mean over time

***Interaction between time and treatment modality

### Relationship between disease duration and improvement degree

**Fig. 3:**
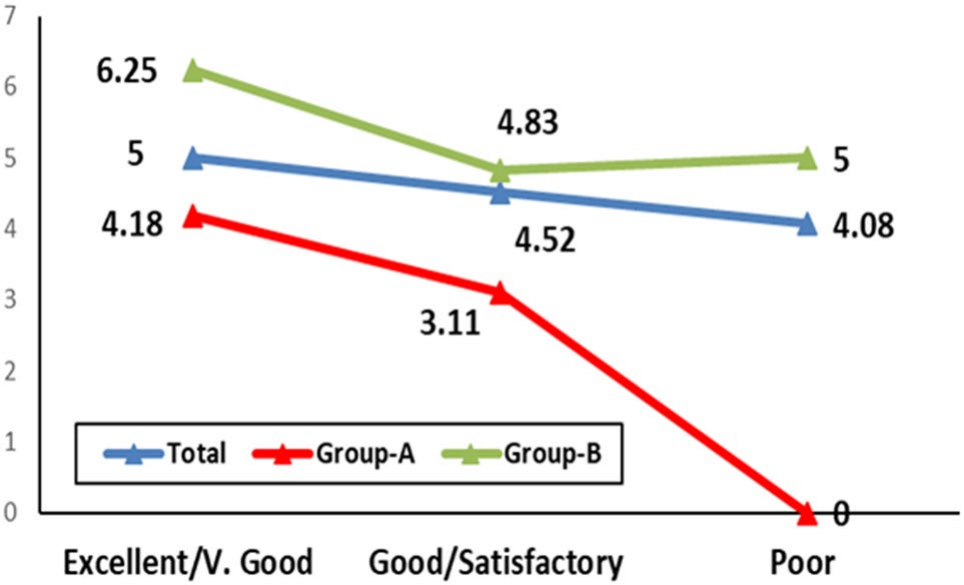
Relationship between disease duration and improvement degree

No effect was detected for the duration of disease on the rate of improvement either for the total sample (p = 0.830), group-A (p = 0.395) or group-B (p = 0.699) (Fig. 3).

### Relationship between lesion sites and improvement degree (Total)

**Fig. 4:**
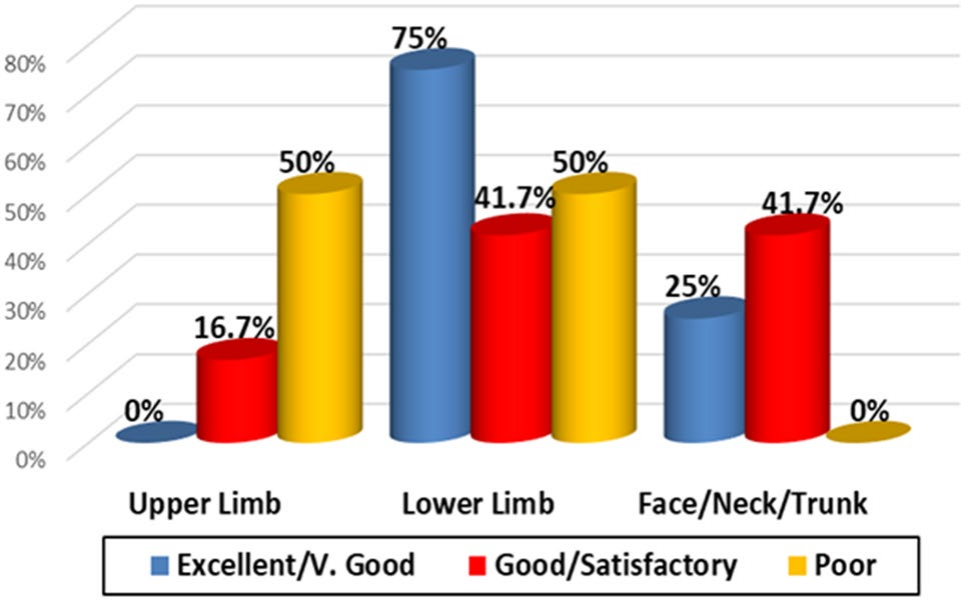
Relationship between lesion site and improvement degree (total)

There was nonsignificant difference between degrees of improvement for lesion sites and improvement degree for total sample (p = 0.397) (Fig. 4).

### Relationship between VASI score before and improvement degree

**Fig. 5:**
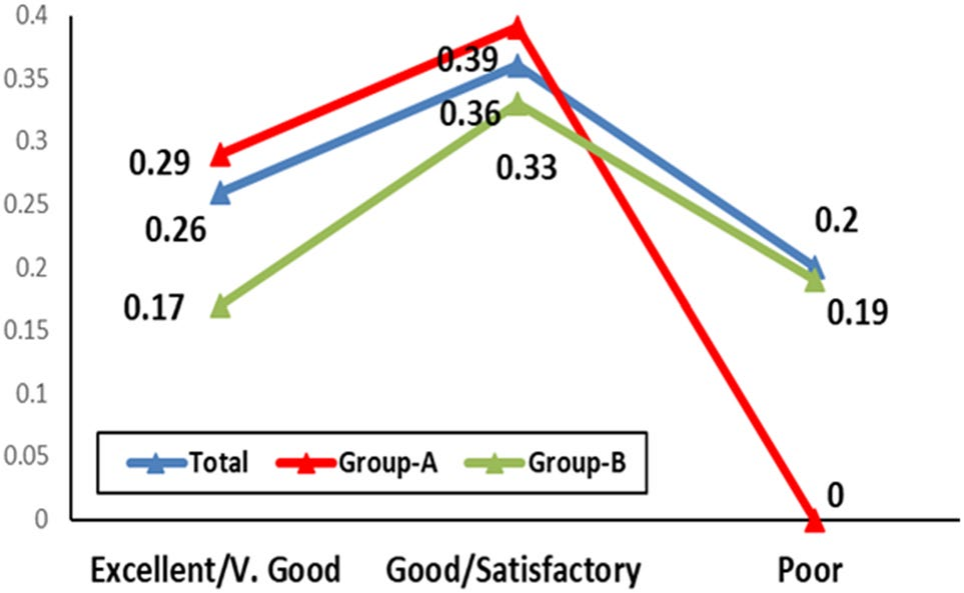
Relationship between VASI score before and improvement degree

Non-significant difference was observed between VASI score before and improvement degree for the total sample and both groups separately (p = 0.635, 0.301 and 0.676) (Fig. 5).

## Discussion

There are various ways to treat vitiligo, but the focus of treatment must be on the clinical presentation and course of the illness. There is currently no one therapy strategy that has been proven to be the most successful in treating vitiligo [16]. Comparing combination therapy to conventional monotherapies, greater rates of repigmentation might result. Therefore, in this study, for the treatment of localized stable vitiligo, we assessed the effectiveness of platelet-rich plasma in conjunction with fractional Er:YAG laser and microneedling.

There was significant difference (p = 0.002) between both treatment modalities for the degree of repigmentation: in fractional Er:YAG + PRP group (A) improvement was observed in 100% (excellent 30%, very good 15%, good 30% and satisfactory 25%) this is comparable to the study by Mokhtari et al. [17] that evaluated the effectiveness of Er:YAG laser in vitiligo patients as an adjuvant treatment to topical 5FU and clobetasol over a 4-month period. The Er:YAG group performed better across all locations. In the treatment group, increases in pigmentation score were seen in 57.4% (very good 21.1%, good 25.8%, and moderate 10.5%) of lesions, compared to 21.1% in the control group. Our better results are possibly attributed to the limited number of sessions in the previous study, the combination effect of fractional Er:YAG and PRP in our study or laser parameters used.

Our fractional Er:YAG + PRP results are considerably better to a study by Al-Dhalimi et al. [18]. Evaluated the potential benefits of using a narrow band ultraviolet B (NB-UVB) and fractional Er:YAG 2940 nm laser combination to treat refractory acral vitiligo. The following response rate was observed in regions treated with both NB-UVB and a fractional Er:YAG laser: compared to 0% in NB-UVB alone, 13.3% had a good reaction, 20% had a moderate response, 23.3% had a poor response, and 43.3% had no response. The statistical analysis showed a significant response (p value < 0.001). This may be explained by the refractory acral vitiligo areas treated, short duration of the latter study (3 months) compared to the current study and the synergistic effect of PRP in our study.

In agreement with our study Abdelghani et al. [19] have demonstrated that fractional CO2 laser and PRP together produced much higher rates of repigmentation than PRP monotherapy. 40% of patients experienced repigmentation above 75%, while 60% of patients experienced repigmentation exceeding 50%. These were considerably better to our results because fractional Er–YAG laser provides less ablation and coagulation depth than fractional CO2 laser, yielding comparable efficacy with lower downtime and less side effects.

These better results achieved in our fractional Er:YAG + PRP group agree also with Botsali and Caliskan [20], who found that patients with stable vitiligo following a typical schedule of twice-weekly high-dose fractional 2940 nm Er:YAG laser uses in addition to several forms of therapy, found that fractional Er:YAG laser is a safe and reliable supplementary treatment. 50% repigmentation was attained in 66.7% of study regions at the third-month control.

In the contrary, our conducted (Mn + PRP) group (B) 80% of cases demonstrated repigmentation (20% very good, 10% good, 50% satisfactory) which is considerably better than research conducted in Egypt by Abdelaty [21]. They found that in the following eight sessions of skin needling, 38.5% of cases treated with PRP showed noticeable repigmentation (moderate response). This may be attributed to different PRP preparation methods, needle depth and density or limited number of sessions.

Like that a study by Attwa et al. [22] showed a significant statistical difference between the effects of 5-FU microneedling and microneedling alone. Only 18.5% of cases had a Mn response (3.7% good and 14.8% satisfactory), compared to 70.4% of cases with 5-FU added (3.7% excellent, 3.7% very good, 11.1% good, and 51.9% satisfactory). These were a similar to our Mn + PRP group results although they used 5-FU over 3-month, study period and we used PRP over 6-month period.

Another study by Ebrahim and Albalate [23] for the treatment of localized stable vitiligo over a 6-month period, there was an approved high statistical difference between the effects of Mn alone and Mn combined with tacrolimus. The Mn response was 56.5% (33.3% excellent, 3.3% very good, 6.6% good, 13.3% satisfied), while the Mn+ tacrolimus response was 93.2% (66.6% excellent, 10% very good, 13.3% good, 3.3% satisfied). This was considerably better than our Mn + PRP group results, possibly due to different needle depth and density and the different topical agent (tacrolimus).

Furthermore, in our study there was significant difference (p = 0.005) between both treatment modalities for the degree of patients’ satisfaction i.e., highly satisfied/satisfied was observed in 45% (30% and 15%) in fractional Er:YAG + PRP group(A) than 15% (0% and 15%) in (Mn + PRP) group(B).This also in favor of fractional Er:YAG and PRP using. Similarly, Ahlawat et al. [24] acknowledged that the PRP and laser group produced higher outcomes in terms of patient satisfaction and repigmentation.

In our study both groups showed no significant difference regarding mean VASI score at baseline (p = 0.571) and after treatment (p = 0.725); however, when comparing the post-treatment VASI with the baseline VASI, there was a significant decrease in the VASI score for both groups [p = 0.001 for group(A) and 0.003 for group(B)]. This indicated that combined Fractional Er:YAG laser + PRP is better than microneedling + PRP which was approved by Mokhtari et al. [17] where Er:YAG group had a larger reduction in patch size (p-value = 0.004). Likewise, there was significant difference for the interaction between time and mode type (p = 0.017) i.e., group(A) had better result for the VASI score (reduction of 16%) compared with group(B) (reduction of 4%), the study by Abdelaty [21]. With a mean reduction of 8.21%, there was a statistically significant decline in the post-treatment cases (skin needling and PRP) compared to pretreatment. This also indicates that fractional Er:YAG + PRP is superior to Mn + PRP in the treatment of stable vitiligo. Non-significant difference was observed between VASI score before treatment and improvement pattern the total sample and both groups separately (p = 0.635, 0.301 and 0.676).

In terms of side effects, there was significantly (p = 0.048) higher rates of side effects among cases treated with Mn + PRP (25%) in the form of pain, which is lower than those in the study by Abdelaty [21]. Whereas burning sensation was one of the side effects in cases treated with fractional YAG + PRP, occurring in 5% of cases, 61.5% of the studied cases reported problems (erythema, burning sensation, and pain during sessions). This was also approved by Al-Dhalimi et al. [18] where no patient receiving laser treatment experienced any localized side effects. Therefore, fractional Er:YAG laser therapy can help in lowering side effects from long-term topical therapies, obtaining a quicker response, and enhancing patient compliance.

There was insignificant difference (p = 0.063) in the duration of disease between groups and the rate of improvement either for the total sample (p = 0.830), fractional Er:YAG + PRP group(A) (p = 0.395) or Mn + PRP group(B) (p = 0.699).This is against Kadry et al. [25] where a strong inverse link, supported by the P-value and correlation coefficient, was discovered between the duration of the disease and proportion of surface area reduction in vitiligo.

Regarding the sites of lesion (fractional Er:YAG + PRP), group(A) had insignificantly (p = 0.525 and 0.548) lower percentage of lower limb, neck and trunk affection compared with (Mn + PRP) group(B). On the other hand, group(A) had an insignificantly (p = 0.197 and 0.376) higher percentage of upper limb and face affection compared with group(B). There was nonsignificant difference between degrees of improvement for lesion sites for total sample (p = 0.397), and each group separately, i.e., groups(A) and (B) (p = 0.258 and 0.140) which is similar to Abdelaty [21]. When the distribution of the disease shows no statistically significant difference between the non-responsive cases and the moderate response, and against Kadry et al. [25], where the facial lesions in the PRP and laser groups responded the best, followed by the acral and truncal lesions. Lesions on the feet and LL had less response. In regions containing hair follicles, the response to therapy is enhanced.

Moreover, re-pigmentation was significantly (p = 0.038) not present in about one-fifth of Mn + PRP group vs no case in Fractional Er:YAG + PRP group, this indicated that Fractional Er:YAG + PRP is superior to Mn + PRP. Also, distribution of re-pigmentation pattern was comparable for the two groups i.e., diffuse (p = 0.744), combined and marginal (p = 0.465) and peri-follicular (p = 0.548); this was against Abdelaty [21], where pigmentation extended from the lesion’s edge to its center, exhibiting a slight reduction in lesion size as opposed to the perifollicular pigmentation typically observed with conventional topical therapies.

### Limitations

Limitations of this study were the small sample size, the lack of consensus regarding preparation methods and laser parameters makes it difficult to compare results from different clinical studies.

## Conclusion

Both treatment modalities could induce repigmentation but combination of fractional Er–YAG laser and PRP demonstrated greater improvement and a satisfactory repigmentation rate compared to PRP with microneedling.

## Supplementary Information

Below is the link to the electronic supplementary material.

Supplementary file1 (DOC 36 KB)

## Data Availability

No datasets were generated or analysed during the current study.
