# Supplementary material for: Effectiveness of fractional erbium–YAG laser, microneedling, platelet-rich plasma in localized stable vitiligo patients: randomized clinical trial
Source: Arch Dermatol Res. 2024 Jun 15;316(7):399. doi: 10.1007/s00403-024-03035-8 (PMC11180153; doi:10.1007/s00403-024-03035-8)
Supplement: Supplementary file 1 — Supplementary file1 (DOC 36 KB) [file 403_2024_3035_MOESM1_ESM.doc]

**CONSORT 2010 Flow Diagram**

**Allocation**

**Analysis**

**Follow-Up**

**Enrollment**

Assessed for eligibility (n=42 )

Excluded (n=2)

♦  Not meeting inclusion criteria (n=1 )

♦  Declined to participate (n=1 )

♦  Other reasons (n=0 )

Analysed (n=20)
♦ Excluded from analysis (give reasons) (n=0)

Lost to follow-up (give reasons) (n=0)

Discontinued intervention (give reasons) (n=0)

Allocated to intervention (n=20)

♦ Received allocated intervention (n=20 )

♦ Did not receive allocated intervention (give reasons) (n=0 )

Lost to follow-up (give reasons) (n=0)

Discontinued intervention (give reasons) (n=0)

Allocated to intervention (n=20)

♦ Received allocated intervention (n=20 )

♦ Did not receive allocated intervention (give reasons) (n=0 )

Analysed (n=20 )
♦ Excluded from analysis (give reasons) (n=0 )

Randomized (n=40 )
